# Evidence for the presence of biogenic magnetic particles in the nocturnal migratory brown planthopper, *Nilaparvata lugens*

**DOI:** 10.1038/srep18771

**Published:** 2016-01-05

**Authors:** Weidong Pan, Guijun Wan, Jingjing Xu, Xiaoming Li, Yuxin Liu, Liping Qi, Fajun Chen

**Affiliations:** 1 Beijing Key Laboratory of Bioelectromagnetics, Institute of Electrical Engineering, Chinese Academy of Sciences, Beijing 100190, China; 2 Department of Entomology, College of Plant Protection, Nanjing Agricultural University, Nanjing 210095, China; 3 Department of Biomedical Engineering, Dalian University of Technology, Dalian 116024, China

## Abstract

Biogenic magnetic particles have been detected in some migratory insects, which implies the basis of magnetoreception mechanism for orientation and navigation. Here, the biogenic magnetic particles in the migratory brown planthopper (BPH), *Nilaparvata lugens* were qualitatively measured by SQUID magnetometry, and their characteristics were further determined by Prussian Blue staining, electron microscopy and energy dispersive x-ray spectroscopy. The results indicate that there were remarkable magnetic materials in the abdomens and not in the head or thorax of the 3^rd^–5^th^ instar nymphs, and in macropterous and brachypterous female and male adults of BPH. The size of magnetic particles was shown to be between 50–450 nm with a shape factor estimate of between 0.8–1.0 for all the tested BPHs. Moreover, the amount of magnetic particles was associated with the developmental stage (the 3^rd^–5^th^ instar), wing form (macropterous vs. brachypterous) and sex. The macropterous female adults had the largest amount of magnetic particles. Although the existence of magnetic particles in the abdomens of BPH provides sound basis for the assumption of magnetic orientation, further behavioral studies and complementary physical characterization experiments should be conducted to determine whether the orientation behavior of BPH is associated with the magnetic particles detected in this study.

Many species of insect have the ability to undertake orientated dispersal or migration. The mechanisms of flight orientation for migratory insects mainly includes sun compass^1^, magnetic compass^2^, celestial cues^3^, polarized light^4^, and compensation for wind drifts and wind orientation^5^. Swarming insects such as desert locusts achieve relatively precise navigation by integrating various orientation cues^6^. Although it has been ascertained that diurnal migratory insects use a time compensated sun compass for their migratory orientation^1^,^7^, the orientation mechanisms of nocturnal migratory insects are still to be explored.

It is well known that many types of insect, such as butterflies, moths, locusts, and dragonflies, undertake impressive seasonal mass migrations travelling up to thousands of kilometers to their ultimate destination^8^,^9^,^10^,^11^. Although biogenic magnetite has previously been shown to be present in monarchs (*Danaus plexippus*), that presence by itself does not constitute solid evidence of magnetic perception. Unlike the sun compass, a magnetic compass does not need to be continuously time compensated. Sensitivity to the magnetic field has been proposed to occur mainly through two non-mutually exclusive mechanisms, that is, the light-dependent chemical-based magnetoreception, and the ferromagnetic particle (magnetite)-based magnetoreception^12^,^13^.

Actually, the magnetite-based mechanism was partly inspired by the production of magnetite crystals by some bacterial that are able to physically move along with geomagnetic field lines^14^. Magnetite was later found in animals that are known to orient with respect to the geomagnetic field^15^. Most magnetite isolated from animals is in the form of single-domain magnetite crystals, which means that they are uniformly and stably magnetized and have the maximum magnetic moment per unit volume possible for magnetite, and the production of this biomineral is likely under precise biological control. These permanently magnetized magnets can twist into alignment with geomagnetic field if allowed to freely rotate. Magnetite crystals in some animals smaller than single-domain size are referred to as superparamagnetic, and they do not have a permanent magnetic moment. However, the magnetic axis of a superparamagnetic crystal tracks the axis of the ambient field, even though the crystal itself remains stationary^16^. These superparamagnetic crystals can generate fields to attract or repel each other. Single domain magnetite and superparamagnetic crystals have both been proposed to function in the magnetoreception of some birds and fish based on anatomical analyses and the use of strong magnetic pulses^17^,^18^,^19^,^20^. Both forms of magnetite crystals are thought to transduce magnetic fields sensory information by interacting with mechanoreceptors of mechanosensitive ion channels when they move to align with magnetic fields^14^,^21^.

On the other hand, the light-dependent chemical-based magnetoreception proposes that an earth-strength magnetic field can influence the product yield of a radical-pair reaction, and thus geomagnetic fields are perceived by chemical reactions involving specialized photoreceptors^13^. Among the reported investigations, the blue/UV light photoreceptor cryptochrome was shown to be essential for light-dependent magnetosensitivity in *Drosophila melanogaster*^22^.

The brown planthopper (BPH), *Nilaparvata lugens* (Stål) (Hemiptera: Delphacidae) was a major rice pest in tropical Asia, and it annually caused severe damage on rice production following the introduction of high-yielding rice varieties in the 1960s^23^,^24^. BPH also vectors rice ragged stunt virus (RRSV) and rice grassy stunt virus (RGSV), causing ragged stunt and grassy stunt disease of rice^25^. BPH is an obligate insect pest of rice crops that does not overwinter in mainland China^26^. It migrates nocturnally from Southeast Asia and across mainland China to Northeast Asia in spring, and back to Southeast Asia in autumn^27^. This pattern of migration raises the questions of how the BPH orients itself during nocturnal flights, and whether a geomagnetic compass is involved?

In our previous study, two novel cryptochrome genes, *Nlcry1 and Nlcry2*, were cloned from the BPH^28^. Quantitative PCR revealed highly variable and distinct cryptochrome expression profiles for both *Nlcry1* and *Nlcry2* across all developmental stages. We also found higher cryptochrome expression levels in the heads of macropterous versus brachypterous individuals^28^. Meanwhile, we have established a manipulative near-zero magnetic field (NZMF) to investigate the potential light-dependent and chemical-based magnetoreception in white-backed planthopper, *Sogatella furcifera,* another species of migratory rice planthopper^29^. We found significantly altered transcript expression level of *cryptochrome*, which is regarded as the main chemical magnetoreceptor.

Given that the cryptochrome-mediated and magnetite-mediated magnetoreception mechanisms are non-mutually exclusive, we conducted a series of experiments to detect the existence of magnetite-based magnetoreception in the BPH. If magnetic particles are found in BPH, considering that only macropterous adults are able to travel long distances, and the body size of females is larger than that of males, we hypothesize that magnetic particles may be more abundant in adults than in nymphs, in female than in male adults, and in macropterous than in brachypterous adults. Thus, given the above assumptions, the existence and nature of magnetic particles in the nymphs, and macro-/brachy-pterous female and male adults of BPH was assessed using SQUID magnetometry, histological staining, transmission electron microscopy and energy dispersive x-ray spectroscopy.

## Results

### Magnetism measurements of the abdomen and cephalothorax of macropterous and brachypterous adults of female and male BPH

**Figure 1:**
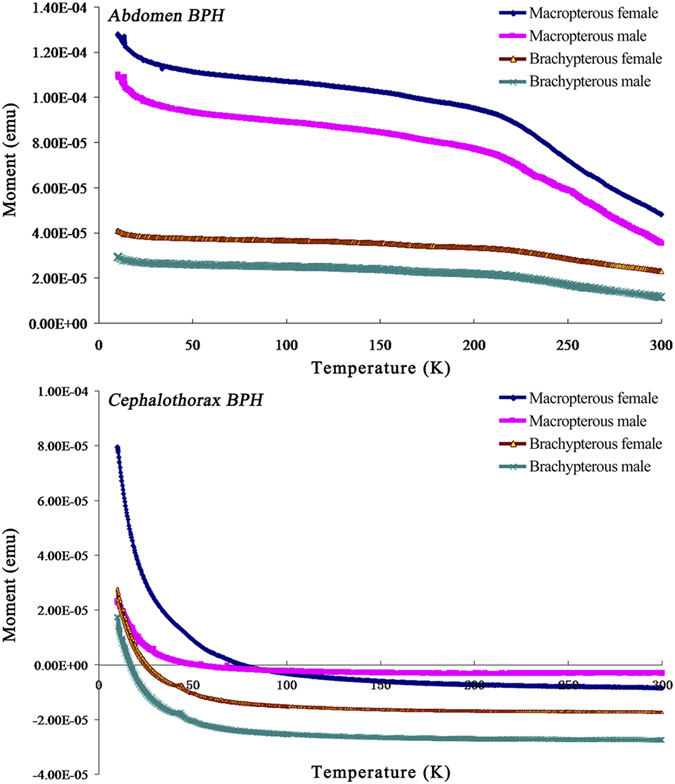
The temperature-demagnetization curves determined from the abdomen and cephalothorax (head+thorax including the antennae) of the macropterous and brachypterous of female and male adult brown planthoppers (BPH), *Nilaparvata lugens*. The determination of temperature-demagnetization curves (T = 10 ~ 300 K) was carried out using a Superconducting Quantum Interference Magnetometer as described in the “Materials and methods”, and the measurements show the process of the test materials retreating to zero from the remanent magnetization. Saturation remanence acquired in a 5T magnetic field at 10 K was demagnetized and measured by warming from 10 to 300 K. Twenty replicate samples of individual abdomen or cephalothorax for either form of adult BPH were used for the magnetic measurements. The arrows denote the turning points of the curves as detected at T = 220 K.

**Figure 2:**
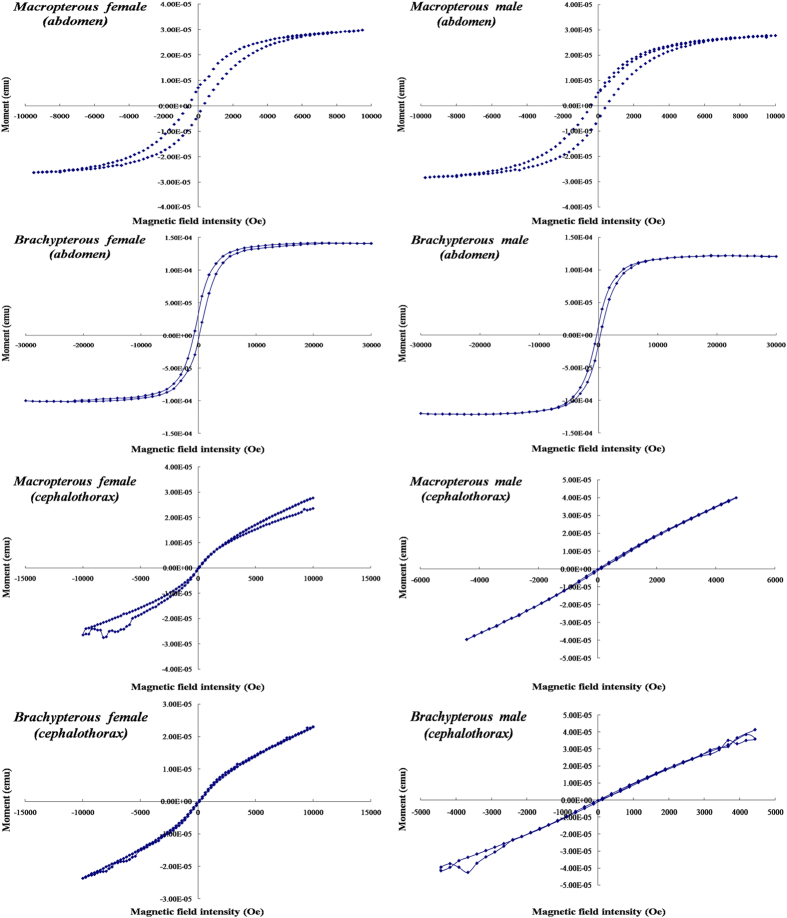
The hysteresis loop determined from the abdomen and cephalothorax (head+thorax including the antennae) of adult BPHs, *Nilaparvata lugens*. The macropterous and brachypterous female and male adults were tested for the presence of magnetic materials in abdomen and cephalothorax. Low-temperature hysteresis loops at T = 10 K were measured between ±3 T with an averaging time of 100 ms using a Superconducting Quantum Interference Magnetometer as described in “Materials and methods”. Twenty replicate samples of individual abdomen or cephalothorax for either form of adult BPH were used for the magnetic measurements.

The temperature demagnetization curves (T = 10–300 K) and hysteresis loop were measured with the abdomen and cephalothorax of macropterous and brachypterous adults of female and male BPH using the SQUID magnetometer. The inflexions of temperature-demagnetization curves in the abdomens of BPH adults were clearly detected at T = 220 K, while no typical curves were detected at T = 220 K for the cephalothorax of BPH adults (Fig. 1). The hysteresis loops were also clearly closed for the abdomens of either macropterous or brachypterous adults (Fig. 2), indicating that hysteresis (i.e., coercive force) and some kind of magnetic materials retained in the abdomens of BPH adults. For cephalothorax parts, however, the magnetism was determined to be weak compared with that in the abdomens, and no closed hysteresis loops were observed in the cephalothorax (heads & thoraces including antennae) of either macropterous or brachypterous BPH adults. On the basis of our initial investigation of magnetism in BPH bodies, we used the abdomens of nymphs, macropterous and brachypterous adults of female and male BPH for further identification of magnetic materials.

### Iron deposits of magnetic material stained in the abdomens of the 1^st^–5^th^ instar nymphs and BPH adults by Prussian Blue staining

**Figure 3:**
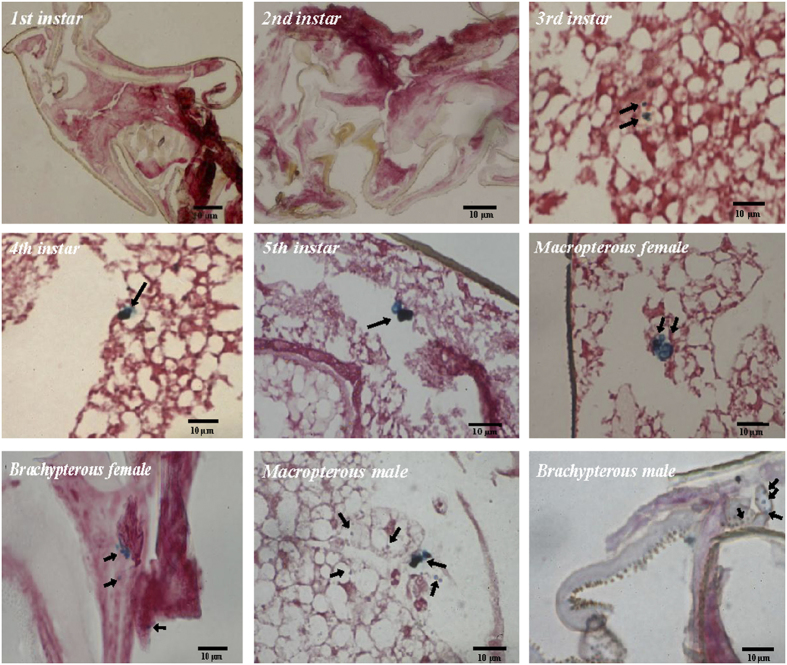
Prussian Blue (PB)-stained sections in the abdomens of the 1^st^–5^th^ instar nymphs, macropterous and brachypterous female and male adult BPHs, *Nilaparvata lugens*. PB-reactive products of high concentrations of iron appear as small pale-blue spherules (arrows) or dark-blue aggregates (arrows). Bar = 10 μm. More than 130 sections were used for counting for each insect stage, sex and wing form. The sections were evaluated under an Olympus microscope BX51 (Olympus, Japan) with a DP70 digital camera.

Figure 3 shows that Prussian Blue staining was observed in the abdomen of both macropterous and brachypterous female and male BPH adults as well as in the 3^rd^–5^th^ instar nymphs, while no staining was detected in either the 1^st^ or the 2^nd^ instar nymph. It is noteworthy that there are two different intracytoplasmic Prussian blue deposits inside the abdomen sections of BPH adults (Fig. 3), that is, small dark-blue stained granules and irregular-shaped dark-blue stained deposits. The observed dark-blue aggregates were similar to those observed and indicative of magnetic particles in the upper beak of homing pigeon^30^,^31^,^32^. More stained granules could be observed in BPH nymphs, whereas more wispy amorphous Prussian blue deposits were observed in BPH adults.

### Fine structure and size distribution analysis of magnetic particles in the abdomens of the 3^rd^–5^th^ instar nymphs and BPH adults by transmission electron microscopy

**Figure 4:**
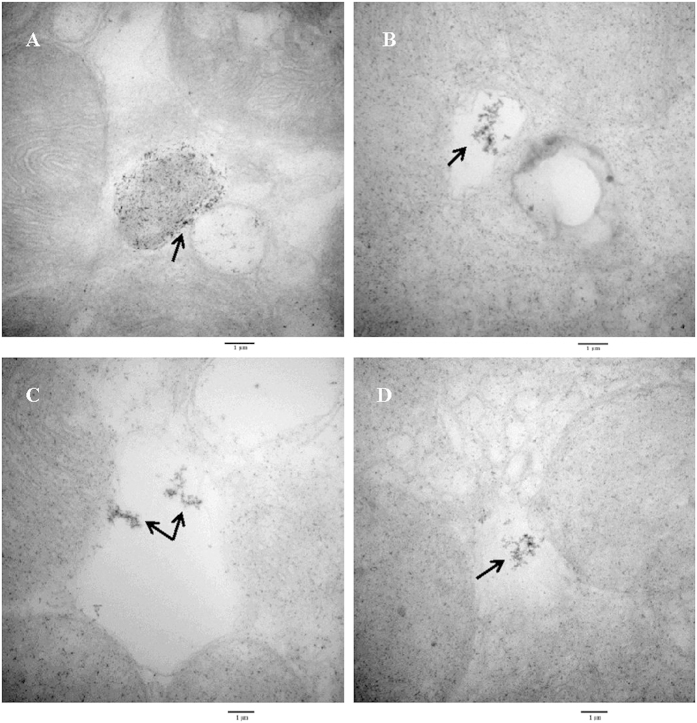
Transmission electron micrographs (TEM) showing fine structures of magnetite particles in the abdomens of macropterous and brachypterous female and male adult of BPHs, *Nilaparvata lugens*. A large magnetite-containing spherule (arrow, dense dotted vesicle) was identified in macropterous female adults (**A**); A magnetite-containing empty vesicle shows iron aggregates (arrow) in macropterous male adults (**B**), brachypterous female adults (**C**), and male adults (**D**). The samples were selected from a series of 35 consecutive ultrathin sections cut out of one unstained semi-thin section between two adjacent Prussian Blue-stained semi-thin sections. More than 150 grids were used for counting for each insect stage, sex and wing form. Bar = 1μm.

**Figure 5:**
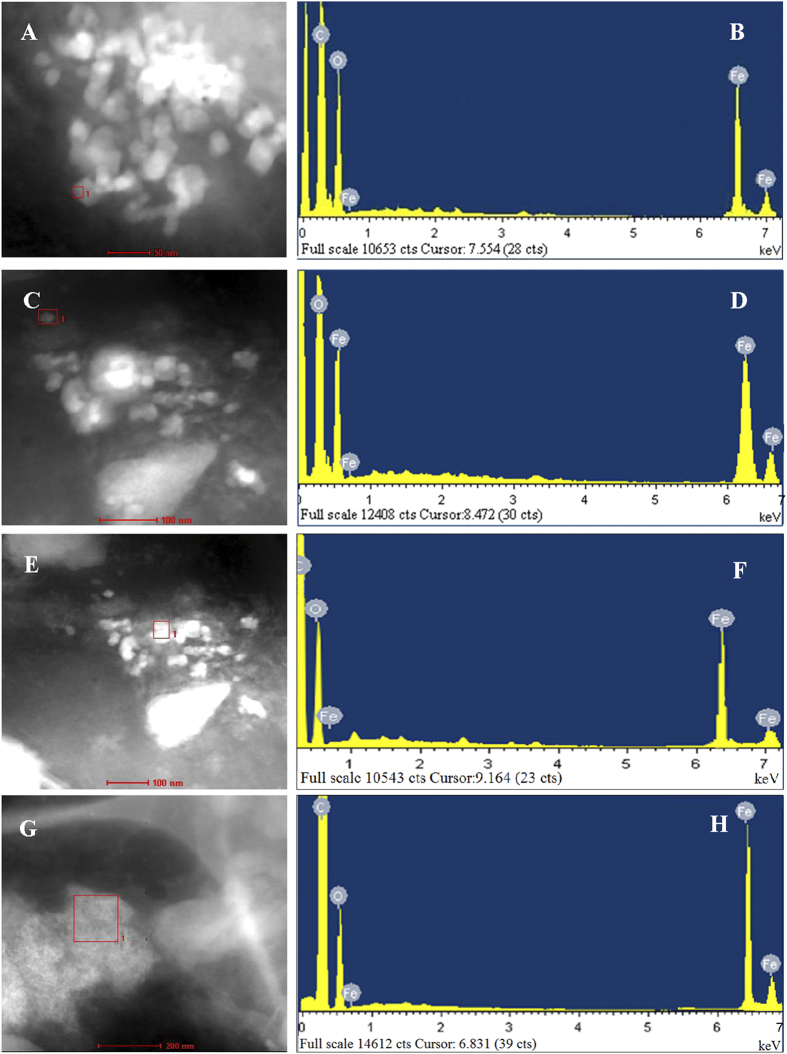
Energy dispersive x-ray (EDX) analysis of isolated magnetic particles from the abdomens of adult BPHs, *Nilaparvata lugens*. (**A,C,E,G**) show transmission electron microscopic images with selected areas (pink boxes) for EDX analysis of macropterous females and males, and brachypterous females and males respectively; (**B,D,F,H**) show the corresponding EDX spectra over the same selected area, respectively. Five hundred adults were used for the analysis.

The abdominal sections described above were selected for further electron microscopic inspection and energy dispersive x-ray (EDX) analysis. The transmission electron micrographs show that magnetite particles or aggregates appeared as two basic features in the pattern (Fig. 4), that is, oval to roundish spherules containing tiny and dotted electron dense particles that were distributed all over, and empty vesicles filled with dense clusters packed in the middle axis of the structure. The EDX analysis shows that either inside the spherule or within the vesicle, iron occurs with oxygen (Fig. 5), indicating a typical component energy spectrum of ferroferric oxide (magnetite).

**Figure 6:**
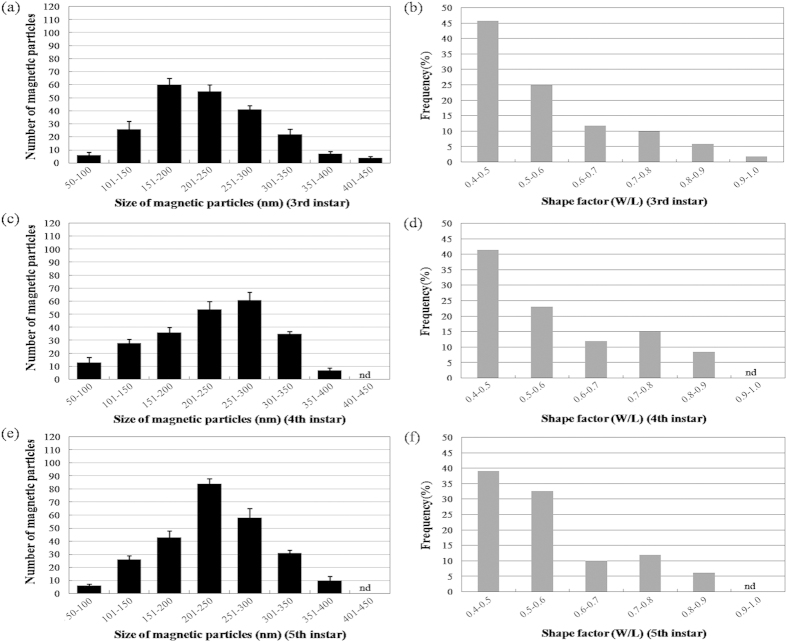
Size distribution and shape factor of magnetic particles calculated from the transmission electron micrographs in nymphs of BPH, *Nilaparvata lugens*. (**a–f**) size distribution and shape factor of the 3^rd^, 4^th^ and 5^th^ instar nymphs with N = 221, 234 and 258, respectively. N, total number of magnetic particles used in this calculation. Five hundred individual insects for each insect stage, sex and wing form were sampled to get the total number of particles. nd - not detected.

**Figure 7:**
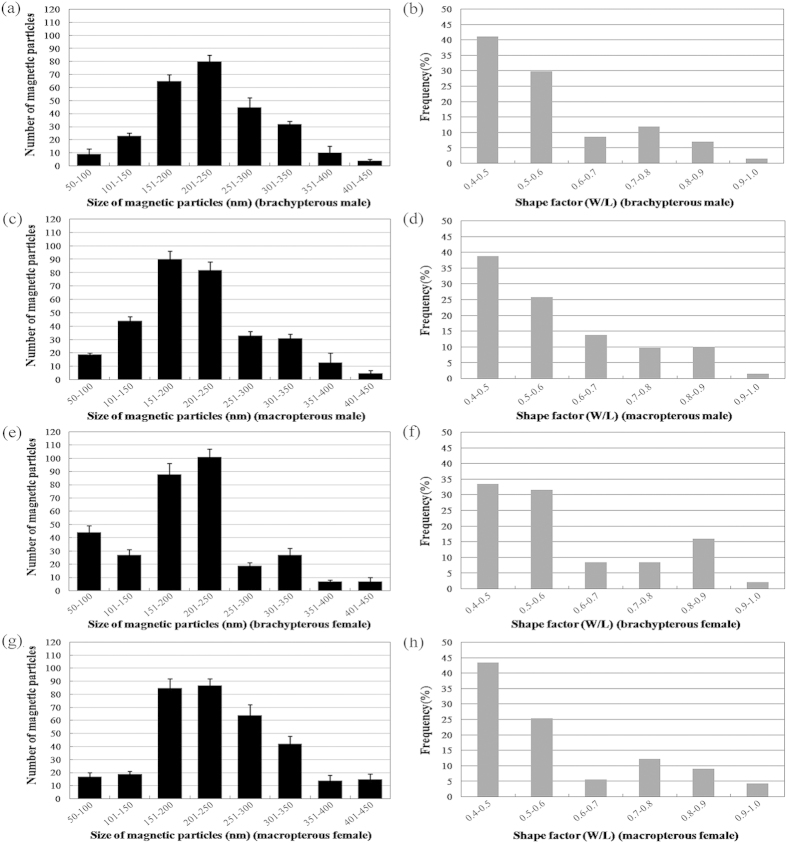
Size distribution and shape factor of magnetic particles calculated from the transmission electron micrographs in adults of BPH, *Nilaparvata lugens*. (**a–h**) size distribution and shape factor of the brachypterous & macropterous male, and brachypterous & macropterous female adults with N = 268, 317, 320 and 343, respectively. N, total number of magnetic particles used in this calculation. Five hundred individual insects for each insect stage, sex and wing form were sampled to get the total number of particles. nd - not detected.

Figures 6 and 7 show the size and shape factor distributions of magnetic particles in the abdomens of the 3^rd^–5^th^ instar nymphs and BPH adults, respectively. The size of magnetic particles in the abdomens was determined to range between 50–450 nm with some of the shape factor between 0.8–1.0 for all developmental periods. The normality test conducted by the Shapiro-Wilk test shows that except in brachypterous and macropterous females, the size of particles in all the other insects corresponds to a normal distribution (3^rd^ instar, *P* = 0.243 > 0.05; 4^th^ instar, *P* = 0.757 > 0.05; 5^th^ instar, *P* = 0.710 > 0.05; brachypterous male, *P* = 0.114 > 0.05; macropterous male, *P* = 0.091 > 0.05; brachypterous female, *P* = 0.026 < 0.05; macropterous female, *P* = 0.040 < 0.05), suggesting the somewhat biogenic uniformity of magnetic particles across the life cycle of BPH (Figs 6 and 7). The size distributions in female adults are different from others, with brachypterous female being indicative of a bimodal distribution (Figs 6 and 7). The shape factor distributions of magnetic particles throughout the insect development were somewhat narrow and asymmetric, also indicating a strictly biochemical control on magnetic particles formation (Figs 6 and 7).

### The relative amount of magnetic particles in the abdomens of the 3^rd^–5^th^ instar nymphs and BPH adults

**Figure 8:**
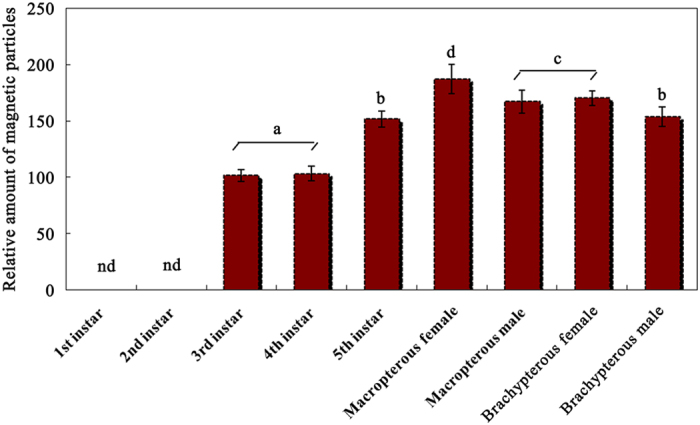
Relative amounts of magnetic particles in the abdomens of BPH, *Nilaparvata lugens*. Prussian Blue staining was used to identify the magnetic particles in the section of abdomens. More than 130 sections were used for counting for each insect stage, sex and wing form. The sections were evaluated under an Olympus microscope BX51 (Olympus, Japan) with a DP70 digital camera. The dark blue stained dots as well as or zones in the images photographed by the digital camera were scored for calculating the relative amount of magnetite using ImageJ software (version 1.47). The mean grayscale values calculated from the dark blue zones of the sections of 3^rd^ instar female insects that contained the lowest amount of magnetic particles were used as a standard (100 percentage ratio) to compare with other values to obtain a percentage ratio to quantify the relative amount of magnetic particles in the abdomens of other instar or adult insects. Different lowercase letters (a–d) indicate significant differences among the tested BPH by LSD test at *P* < 0.05; nd - not detected.

Figure 8 shows the relative amount of magnetic particles determined in the abdomens of the 3^rd^–5^th^ instar nymphs and of female and male BPH. The macropterous female adults contained the greatest amount of magnetic particles, and the 3^rd^ instar nymphs contained the least amount of magnetic particles (Fig. 8). There were significant differences in the relative amount of magnetic particles in the abdomens among the 3^rd^–5^th^ nymphal instar (*P* < 0.01), between macropterous and brachypterous adults (*P* < 0.05) and between female and male adults (*P* < 0.05) (Table 1). No significant effects of the interaction between wing form and sex were found (*P* > 0.05) (Table 1). The results show that the relative amount of magnetic particles in the abdomens of nymphs increased from instar to instar, and female adults showed more magnetite than male adults as well as macropterous adults showed more magnetite than brachypterous adults. These results indicate that the presence of magnetic particles in the abdomens of BPH followed a dynamic process throughout their development.

## Discussion

Previous magnetic measurements of whole insects and body parts including abdomens have shown the presence of superparamagnetic and larger magnetic particles or aggregates in *Apis mellifera* bee^33^,^34^. In the stingless bee *Scaptotrigona postica*^35^, iron granules were observed within size range of 40–160 nm while ferritin-like particles measuring 2.1 ± 0.5 nm were identified in the abdomens^36^. Moreover, magnetic nanoparticles in the *Neocapritermes opacus* termite were estimated to be 18.5 ± 0.3 nm in diameter^37^ and magnetite particles of size distribution around 10 nm were found in the Australian termite *Nasutitermes* and *Amitermes*^38^. In this study, we detected the presence of biogenic magnetic particles, probably magnetite, in brown planthopper (BPH), *Nilaparvata lugens*. To our knowledge, this is the first reported evidence of biogenic magnetic material existing in BPH, a major migratory rice pest, providing important implications for the study of the association between magnetoreception receptor and migratory behaviors.

The Verwey transition appears in the magnetization curves of single-domain or multi-domain magnetite at 110–120 K, and thus can help quickly and accurately identify magnetite in minerals^39^. However, it is difficult to observe the Verwey transition in needle-shaped single-domain magnetite^40^. The internal and external pressures for samples, interactions between domain walls, non-stoichiometry and low temperature oxidation of magnetic particles may suppress the Verwey transition^41^,^42^. The inflexions of temperature-demagnetization curves at T = 220 K were observed in the abdomens of BPH adults and not in the cephalothorax of BPH adults. So far there are no reports about the biological significance of these inflexions at T = 220 K, thus we assume the observed inflexion as an uncharacteristic form of extra transition in the biogenic magnetism of BPH. Different size distributions of magnetic particles were observed in different developmental stages of BPH and a normal size distribution was observed in BPH except with the female adults. The size of magnetic particles in the abdomens was determined to range between 50–450 nm with some of the shape factor between 0.8–1.0 for all developmental periods, indicating that at least some of the particles belong to the single-domain region.

The report on the presence of magnetic particles in the head, thorax and abdomen of migratory ant *Pachycondyla marginata* showed that the size distribution of particles in the abdomen corresponded to a single distribution while a bimodal splitting distribution occurred in the thorax and the head^43^. Due to the absence of magnetism in the head & thorax and the presence of magnetism in the abdomen of BPH with the preliminary investigation by the magnetic measurements, we used the abdomen to further localize the existence of magnetic materials. Compared with the particles in the abdomens of the ant *P. marginata*, a pattern of either normal or bimodal size distribution was observed across the life cycle of BPH, suggesting that the different size distribution of magnetic particles may be related to different functions in their biomineralization.

The magnetic particles present in the abdomen sections of the 3^rd^–5^th^ instar nymphs and BPH adults were determined by Prussian Blue technique modified to stain specifically for magnetite, i.e., when buffered to pH 7.3 in sodium dithionite-citrate solution, magnetite would be the probable iron mineral that stained with Prussian Blue^44^. To our knowledge, this is the first systematic study on the distribution of magnetic materials at all developmental stages of rice planthopper. Previous reports on the characterization of superparamagnetic magnetite in iron granules formed in the trophocytes of honeybee *A. mellifera* by EDX spectra showed the presence of phosphorus, calcium and iron peaks^45^,^46^,^47^. In another species of bee *Scaptotrigona postica*, similar elements including magnesium were present except in different proportions^36^. As iron granules in honeybees only chelate iron and calcium, it was speculated that phosphorus in honeybees may serve as energy sources for ATP synthesis rather than function as a chelator to reduce the toxicity of metal ions^48^. High level of phosphate would prevent the crystallization of iron oxides in the corresponding structures, depriving them of the possibility of participation in magnetosensitivity mechanisms. In this study, the EDX spectra revealed the presence of one iron and three oxygen peaks, which confirmed the nature of ferroferric oxide (magnetite) with the particles. Additionally, carbon was shown to exist either inside the spherule or within the vesicle. As being fundamental to the development and growth of insects, carbon metabolites were surmised to play a key role in the biogenesis of magnetic particles in BPH.

BPH is an insect pest of rice with an r-strategy. The species escapes deteriorated food resources through a long-distance migration in Asia^49^. In a migrant swarm, female BPH with developed ovaries settle first and do not take off again. Consequently, the proportion of male adults increases with migration distance, so that male adults always predominated over females in the population caught on a site far from the source land^50^. As macropterous and brachypterous females are bigger in body size than macropterous and brachypterous males, respectively, the differences in magnetic materials reported between sexes may be explained by differences in body size between sexes (Table 1 and Fig. 8) if bigger insects have more magnetite. However, as macropterous males have similar amount of magnetic particles as brachypterous females (Fig. 8), the differences in magnetic materials may possibly not only relate to sex but also relate to the wing form. Wing dimorphism, i.e., brachypterous versus macropterous insects, is affected by various environmental cues^49^,^51^, and two insulin receptors are proved to determine alternative wing morphs in BPH^51^. The brachypterous individuals always predominate over ones until the nutritional conditions of the rice sheath on which the BPH feeds begin to deteriorate^49^. As the rice plant ages, most of the BPH population becomes macropterous and emigrates, and only a small number of brachypterous insects remains or disperses a short distance locally^49^. From our study, macropterous adults showed more magnetic particles than brachypterous adults. As macropterous insects depend more on flying capacities and orientation, the differences in the amount of magnetic materials observed between macropterous and brachypterous BPHs may provide support for the proposed magnetite-based mechanism for flying orientation.

The accumulation of magnetic particles during instar development of BPH appears to be associated with age. In *Solenopsis* ants, the magnetic material amount of body parts varied from late summer (March) to early winter (July)^52^. The amount of magnetic material was greatest in March and least in June^52^. In addition, *S. richteri* majors presented more magnetic material than minor workers. It thus suggests that the magnetic material amount is size-correlated and probably age-correlated in *Solenopsis*^52^. In honey bee drones, iron granules are reported, but the concentration appears to be age-related^53^. Oenocytes of drones only 0- and 3-days old did not stain positively for iron, while those of 6-, 9- and 12-days did. These results indicate an age-related accumulation of iron related to maturity and/or possibly to biological functionality^53^. Besides being sexually immature, nymphs of BPH may be incapable of directional flight until sufficient magnetic material has been accumulated in the body through the adult stages for the flight orientation during long-distance migration.

BPH accomplishes the seasonal northward and southward migration across mainland China with an average migrating speed of 10 m/s^54^. The direction of migration depends mostly on the seasonal monsoon and happens occasionally at an angle between heading and wind direction^55^. In honeybee *A. mellifera*, magnetite particles found in the abdomens were suggested to be involved in magnetic orientation^56^, and different sizes of crystals were speculated to be related to different functions in magnetic field detection. It has been proposed that single-domain magnetite particles in honeybees determined their compass sense while superparamagnetic crystals were involved in the learned navigational map sense of magnetic intensity gradients^57^. The single domain particles in insect body would more naturally provide angular information than superparamagnetic crystals due to its own magnetization direction with respect to the body. Although the magnetic particles in BPH were found partially to fit the single domain region, further behavioral studies and complementary physical characterization experiments should be performed to determine whether orientation behavior is associated with the magnetite crystals detected in the abdomens of BPH.

In summary, this study provides solid evidence of biogenic compound formation in a migratory rice pest, the BPH, with unidimensional magnetic particles detected in the abdomens. Although the size of the particles partially belongs to the single-domain region regardless of the developmental stages of the insects, the magnetic particles underwent a dynamic change through the life cycle of BPH. Not only was the amount of magnetic particles associated with the developmental stage, but also with the wing form and sex. The recent whole genome sequencing of BPH provides an excellent opportunity for further investigating the molecular mechanism of magnetite-based magnetoreception^58^. In particular, potential genes homologous with magnetosome-assembling genes in magnetotactic bacteria may help in elucidating the biogenesis patterns of magnetic particles in the body of BPH^59^. Thus, the abdomens containing the biogenic magnetic particles may be considered an ideal platform for the exploration of primary processes of possible magnetoreception in BPH.

## Methods

### Insect stocks and pre-treatments

In September of 2013, the 1^st^–5^th^ instar nymphs, macropterous and brachypterous adults of female and male BPHs used in this study were collected from rice fields of Nanjing, Jiangsu Province, China. To avoid the possibility of iron accumulation through diet in abdomen of *Nilaparvata lugens*, the insects were individually isolated according to the methods of Hanzlik *et al.*^31^ using non-magnetic titanium blades and forceps to avoid iron contamination and starved for one day to evacuate the gut of potential iron-containing materials before putting them into the absolute ethyl alcohol for fixation. To avoid magnetic contamination that might cause spurious readings, the insects were prepared by discarding their wings, legs and scales. Furthermore, the abdomens were pressed by non-magnetic tools and washed thoroughly with distilled water before the SQUID measurements. Due to the magnetic weakness of the insect samples, we minimized any magnetic influence of the sample container by using a new sample container with remanence less than 5 × 10^−8^ emu for each sample.

### Magnetism measurements of macropterous and brachypterous female and male adults

The Macropterous & brachypterous female and male BPH adults were used for the magnetism measurements. Because the brown planthopper is very tiny, we used the abdomen and cephalothorax (head+thorax) to investigate the presence of magnetic materials. The antennae were not removed with the other appendages and tested as a whole with the heads and thoraces. Twenty replicate samples of individual abdomen or cephalothorax for either form of adult BPH were used for the magnetic measurements. Magnetism measurements were carried out using a Superconducting Quantum Interference Magnetometer (model MPMS-7, Quantum Design, USA) with a measurement range of 1 × 10^−10^ Am^2^ to 0.3 Am^2^ and with an absolute sensitivity of 1 × 10^−10^ Am (field strength 0.8 MA/m) and 5 × 10^−10^ Am (field strength 4.0 MA/m). The magnetic field range was ±4.8 MA/m within a temperature range of 1.9 K–400 K and sample space <Φ6 mm × 6 mm. The measurements included the determination of temperature-demagnetization curves (T = 10–300 K) and the determination of hysteresis loop (T = 10 K), in which the former described the process of retreating to zero from the remanent magnetization and the latter described the relationship between magnetic flux density or magnetization with magnetic field strength. Saturation remanence acquired in a 5T magnetic field at 10 K was demagnetized and measured by warming from 10 to 300 K. Low-temperature hysteresis loops at T = 10 K were measured between ±3 T with an averaging time of 100 ms.

### Histological examination with Prussian Blue staining and magnetic material amount determination

The abdomens of the 1^st^–5^th^ instar nymphs, macropterous and brachypterous adults of female and male BPH were individually fixed with glutaraldehyde and embedded in paraffin wax. Serial horizontal and sagittal sections of 5 μm thickness were cut using a Leica rotary microtome (RM2126) and mounted directly on microscope slides. In order to localize the magnetic materials, Prussian Blue staining was used to identify the iron magnetic particles. Magnetite (Fe_3_O_4_) is the probable iron mineral that stains with Prussian Blue when buffered to pH 7.3 in sodium dithionite-citrate solution^44^. In the presence of Fe_3_O_4_ and HCl, potassium hexacyanoferrate turns into the dark-blue ferric ferrocyanide. The Prussian Blue was applied for 10 min to yield a clearly visible reaction. After the Prussian Blue reaction, these treated sections were counterstained with kernechtrot^60^ for histological recognition of cellular nuclei. More than 130 sections were used for counting for each insect stage, sex and wing form. The sections were evaluated under an Olympus microscope BX51 (Olympus, Japan) with a DP70 digital camera. The dark blue stained dots as well as zones in the images photographed by the digital camera were scored for calculating the relative amount of magnetite using ImageJ software (version 1.47). The mean grayscale values calculated from the dark blue zones of the sections of 3^rd^ instar female insects that contained the lowest amount of magnetic particles were used as a standard (100 percentage ratio) to compare with other values to obtain a percentage ratio to quantify the relative amount of magnetic particles in the abdomens of other instar or adult insects.

### Transmission Electron microscopy for particle size measurements

Transmission electron microscopy (TEM, Hitachi 7650B) was used further to analyze the iron rich granules. The abdomens of adult BPH were fixed in a pre-cooled 4% paraformaldehyde (0.1M phosphate buffer, pH 7.4) for 24 h, and the fixative was replaced once during the process when needed. After being rinsed in tap water consecutively for 12 h, the treated tissue was placed in a tissue processor for overnight processing by serial alcohol solutions. This was followed sequentially by hyalinization using xylene and by paraffin embedding using an automated embedding station. Some sections in thickness of 60 nm–90 nm were cut with a diamond knife, stained with uranyl acetate and lead citrate, and examined by TEM (Hitachi H600, Japan) operating at an accelerated voltage of 100 kV. More than 150 grids were used for counting for each insect stage, sex and wing form. The size of magnetic particles was measured on TEM micrographs and the size distribution was also analyzed by ImageJ software (version 1.47). The particle sizes were determined by measuring the major (length, L) and minor (width, W) axes of the best-fitting ellipse of the TEM micrographs. The particle size was defined as (L + W)/2, and the shape factor as W/L.

### Purification of magnetic particles and HRTEM for energy dispersive x-ray spectroscopy

Approximately 500 nymphs of each instar or adults were collected, anesthetized with ice, washed with ice-distilled water three times, and dissected with non-magnetic tools. Ventral abdomens were obtained and placed into a 50 ml centrifuge tube, ground with liquid nitrogen and homogenized in 20 ml HEPES buffer (10 mM, pH 7.4). The homogenates were centrifuged at 10,000 × g for 10 min to remove unhomogenized insects. The pellet obtained was suspended in 20 ml HEPES buffer (10 mM, pH 7.4). The suspension was placed in a 50 ml falcon tube and a neodymium boron (Nd-B) magnet (25 × 25 × 12 mm) was stuck on the wall of tube with the distance 10 cm from the bottom (200 mT field intensity at the center of the magnet). The suspension was treated magnetically for 10 h and then the nonmagnetic fluid was removed by aspiration. The collected magnetic particles were washed with 10 mM HEPES buffer (pH 7.4) at least 10 times with slight sonication. This procedure with the magnet was repeated three times, and the purified magnetites were finally collected by centrifugation at 10, 000 × g for 20 min. The precipitate was suspended in 10 mM HEPES buffer (pH 7.4) and stored at −80 °C. All purification steps were conducted at 4 °C. Each treatment of instar nymphs or adults was replicated three times for measurements.

To prepare the specimen for high resolution transmission electron microscopy (HRTEM) observation, a drop of the purified magnetic particles suspension was applied to carbon-coated copper grids and dried at room temperature for about 5 min. After being rinsed three times with PBS for 1 min each time, the grids were rinsed with deionized water twice for 3 min each time. The elemental composition in selected areas of HRTEM observation were analyzed by energy-dispersive x-ray (EDX) spectroscopy in a Tecnai F30 HRTEM operating at an accelerated voltage of 300 kV.

### Statistical analysis

All data were analyzed using the software SPSS 20.0. The relative amount of magnetic particles presented in the abdomens of the 3^rd^–4^th^ instar nymphs of BPH were analyzed by one-way analysis of variances (ANOVAs). Moreover, two-way ANOVAs were also used to analyze the effects of wing dimorphism (macropterous vs. brachypterous), sex and their interactions on the relative amount of magnetic particles presented in the abdomens of macropterous and brachypterous adults of female and male BPH. If significant effects on the above measured indexes were found, the least significant difference (LSD) test was further used to compare the means between treatments at *P* < 0.05. A normality test was conducted for the magnetic particle size distribution by the Shapiro-Wilk test. The least significant difference (LSD) test was further used to compare the means between different size at *P* < 0.05.

## Additional Information

**How to cite this article**: Pan, W. *et al.* Evidence for the presence of biogenic magnetic particles in the nocturnal migratory brown planthopper, *Nilaparvata lugens*. *Sci. Rep.* **6**, 18771; doi: 10.1038/srep18771 (2016).

## Figures and Tables

**Table 1:** One-way analysis of variance (ANOVA) (F/*P* values) with nymph instar (NI) and two-way ANOVAs with wing dimorphism (WD) as main factor (macropterous vs. brachypterous) and sex as sub-factor on relative amounts of magnetite measured in the abdomens of BPH, *Nilaparvata lugens*.

Measured index	NI^a^	WD^b^	Sex	WD × Sex
Relative amount of magnetite	137.01 (0.002)*	6.98(0.020)*	4.87 (0.035)*	3.36 (0.51)

^a^NI - The 3^rd^ to 5^th^ instar.

^b^WD - Macropterous vs. brachypterous.

^*^P < 0.05,

^**^P < 0.01.

## References

[b1] Oliveira E. G., Srygley R. B. & Dudley R. Do neotropical migrant butterflies navigate using a solar compass? J. Exp. Biol. 201, 3317–3331 (1998). 9817829 10.1242/jeb.201.24.3317

[b2] Banks A. N. & Srygley R. B. Orientation by magnetic field in leaf-cutter ants, *Atta colombica* (Hymenoptera: Formicidae). Ethology 109, 835–846 (2003).

[b3] Wehner R. Astronavigation in insects. Annu. Rev. Entomol. 29, 277–298 (1984).

[b4] Rössel S. & Wehner R. Polarization vision in bees. Nature 323, 128–131 (1986).

[b5] Preiss R. & Gewecke M. Compensation of visually simulated wind drift in the swarming flight of the desert locust (*Schistocerca gregaria*). J. Exp. Biol. 157, 461–481 (1991).

[b6] Collett M., Collett T. S., Bisch S. & Wehner R. Local and global vectors in desert ant navigation. Nature 394, 269–272 (1998).

[b7] Perez S. M., Taylor O. R. & Jander R. A sun compass in monarch butterflies. Nature 387, 29 (1997).

[b8] Kennedy J. S. The migration of the desert locust (*Schistocerca gregaria* Forsk.). I. The behaviour of swarms. II. A theory of long-range migrations. Philos. Trans. R. Soc. Lond., B, Biol. Sci. 235, 163–290 (1951). 24541037 10.1098/rstb.1951.0003

[b9] Mouritsen H. & Frost B. J. Virtual migration in tethered flying monarch butterflies reveals their orientation mechanisms. Proc. Natl. Acad. Sci. USA 99, 10162–10166 (2002). 12107283 10.1073/pnas.152137299 PMC126641

[b10] Dingle H. Migration: The Biology of Life on the Move. (Oxford University Press, 1996).

[b11] Wikelski M. *et al.* Simple rules guide dragonfly migration. Biol. Lett. 2, 325–329 (2006). 17148394 10.1098/rsbl.2006.0487 PMC1686212

[b12] Kirschvink J. L., Walker M. M. & Diebel C. E. Magnetite-based magnetoreception. Curr. Opin. Neurobiol. 11, 462–467 (2001). 11502393 10.1016/s0959-4388(00)00235-x

[b13] Ritz T., Adem S. & Schulten K. A model for photoreceptor-based magnetoreception in birds. Biophys. J. 78, 707–718 (2000). 10653784 10.1016/S0006-3495(00)76629-X PMC1300674

[b14] Lohmann K. J. Q&A: Animal behaviour: Magnetic-field perception. Nature 464, 1140–1142, 10.1038/4641140a (2010). 20414302

[b15] Kirschvink J. L. Magnetite Biomineralization and Geomagnetic Sensitivity in Higher Animals - an Update and Recommendations for Future Study. Bioelectromagnetics 10, 239–259, 10.1002/bem.2250100304 (1989). 2665750

[b16] Johnsen S. & Lohmann K. J. The physics and neurobiology of magnetoreception. Nat. Rev. Neurosci. 6, 703–712, 10.1038/nrn1745 (2005). 16100517

[b17] Losos J. B., Schoener T. W. & Spiller D. A. Predator-induced behaviour shifts and natural selection in field-experimental lizard populations. Nature 432, 505–508, 10.1038/nature03039 (2004). 15565155

[b18] Eder S. H. *et al.* Magnetic characterization of isolated candidate vertebrate magnetoreceptor cells. Proceedings of the National Academy of Sciences of the United States of America 109, 12022–12027, 10.1073/pnas.1205653109 (2012). 22778440 PMC3409731

[b19] Semm P. & Beason R. C. Responses to small magnetic variations by the trigeminal system of the bobolink. Brain Res. Bull. 25, 735–740, http://dx.doi.org/10.1016/0361-9230(90)90051-Z (1990). 2289162 10.1016/0361-9230(90)90051-z

[b20] Beason R. C., Dussourd N. & Deutschlander M. E. Behavioral Evidence for the Use of Magnetic Material in Magnetoreception by a Migratory Bird. Journal of Experimental Biology 198, 141–146 (1995). 9317510 10.1242/jeb.198.1.141

[b21] Davila A. F., Fleissner G., Winklhofer M. & Petersen N. A new model for a magnetoreceptor in homing pigeons based on interacting clusters of superparamagnetic magnetite. Physics and Chemistry of the Earth, Parts A/B/C 28, 647–652, 10.1016/s1474-7065(03)00118-9 (2003).

[b22] Gegear R. J., Casselman A., Waddell S. & Reppert S. M. Cryptochrome mediates light-dependent magnetosensitivity in *Drosophila*. Nature 454, 1014–1019 (2008) 18641630 10.1038/nature07183 PMC2559964

[b23] Gallagher K. D., Kenmore P. E. & Sogawa K. In Planthoppers (eds Denno Robert F & Perfect T. John ) Ch. 17, 599–614 (Springer US, 1994).

[b24] Way M. & Heong K. The role of biodiversity in the dynamics and management of insect pests of tropical irrigated rice-a review. Bull. Entomol. Res. 84, 567–588 (1994).

[b25] Hibino H. Biology and epidemiology of rice viruses. Annu. Rev. Phytopathol. 34, 249–274 (1996). 15012543 10.1146/annurev.phyto.34.1.249

[b26] Qi H., Jiang C., Zhang Y., Yang X. & Cheng D. Radar observations of the seasonal migration of brown planthopper (*Nilaparvata lugens* Stål) in Southern China. Bull. Entomol. Res. 104, 731–741 (2014). 25229712 10.1017/S0007485314000558

[b27] Riley J. R. *et al.* The long-distance migration of *Nilaparvata lugens* (Stål) (Delphacidae) in China: radar observations of mass return flight in the autumn. Ecol. Entomol. 16, 471–489 (1991).

[b28] Xu J. J. *et al.* Molecular characterization, tissue and developmental expression profiles of cryptochrome genes in wing dimorphic brown planthoppers, Nilaparvata lugens. Insect Sci. 10.1111/1744-7917.12256 (2015). 26227859

[b29] Wan G. J. *et al.* Cryptochromes and hormone signal transduction under near-zero magnetic fields: new clues to magnetoreception in a rice planthopper. PLoS ONE, 10.1371/journal.pone.0132966 (2015). PMC4501744 26173003

[b30] Fleissner G. *et al.* Ultrastructural analysis of a putative magnetoreceptor in the beak of homing pigeons. J. Comp. Neurol. 458, 350–360 (2003). 12619070 10.1002/cne.10579

[b31] Hanzlik M. *et al.* Superparamagnetic magnetite in the upper beak tissue of homing pigeons. Biometals 13, 325–331 (2000). 11247039 10.1023/a:1009214526685

[b32] Winklhofer M., Holtkamp-Rötzler E., Hanzlik M., Fleissner G. & Petersen N. Clusters of superparamagnetic magnetite particles in the upper-beak skin of homing pigeons: Evidence of a magnetoreceptor? Eur. J. Mineral. 13, 659–669 (2001).

[b33] Takagi S. Paramagnetism of honeybees. J. Physical. Soc. Japan 64, 4378–4381 (1995).

[b34] El-Jaick L. J., Acosta-Avalos D., Motta de Souza Esquivel D., Wajnberg E. & Paixão Linhares M. Electron paramagnetic resonance study of honeybee *Apis mellifera* abdomens. Eur. Biophys. J. 29, 579–586 (2001). 11288833 10.1007/s002490000115

[b35] Cunha M. A. S., Walcott B. & Sesso A. Iron-containing cells in the stingless bee *Scaptotrigona postica* Latreille (Hymenoptera: Apidae). Morphology and ultrastructure. In Eder J., Rembold H., eds., Chemistry and Biology of Social Insects. Verlag; Munchen: pp. 91 (1987).

[b36] Keim C. N., Cruz-Landim C., Carneiro F. G. & Farina M. Ferritin in iron containing granules from the fat body of the honeybees *Apis mellifera* and *Scaptotrigona postica*. Micron 33, 53–59 (2002). 11473814 10.1016/s0968-4328(00)00071-8

[b37] Alves O. C., Wajnberg E., De Oliveira J. F. & Esquivel D. M. Magnetic material arrangement in oriented termites: a magnetic resonance study. J. Magn. Reson. 168, 246–251 (2004). 15140434 10.1016/j.jmr.2004.03.010

[b38] Maher B. A. Magnetite biomineralization in termites. Proc. R. Soc. Lond., B, Biol. Sci. 265, 733–737 (1998).

[b39] Verwey E. J. W. Electronic conduction of magnetite (Fe_3_O_4_) and its transition point at low temperatures. Nature 144, 327–328 (1939).

[b40] Muxworthy A. R. Low-temperature susceptibility and hysteresis of magnetite. Earth Planet. Sci. Lett. 169, 51–58 (1999).

[b41] Moskowitz B. M., Jackson M. & Kissel C. Low-temperature magnetic behavior of titanom agnetites. Earth Planet. Sci. Lett. 157, 141–149 (1998).

[b42] Muxworthy A. R. & McClelland E. The causes of low-temperature demagnetization of remanence in multidomain magnetite. Geophys. J. Int. 140, 115–131 (2000).

[b43] Acosta-Avalos D. *et al.* Isolation of magnetic nanoparticles from *Pachycondyla marginata* ants. J. Exp. Biol. 202, 2687–2692 (1999). 10482727 10.1242/jeb.202.19.2687

[b44] Kirschvink J. L. *et al.* Magnetite-based magnetoreceptor cells in the olfactory organ of rainbow trout and zebrafish. In *American Geophysical Union, Fall Meeting 2011*, San Francisco. abstract #B52A-04 (2011).

[b45] Kuterbach D. A., Walcott B. R., Reeder J. & Frankel R. B. Iron-containing cells in the honey bee (*Apis mellifera*). Science 218, 695–697 (1982). 17791591 10.1126/science.218.4573.695

[b46] Kuterbach D. A. & Walcott B. R. Iron-containing cells in the honeybee (*Apis mellifera*). I. Adult morphology and physiology. J. Exp. Biol. 126, 375–387 (1986). 3805998 10.1242/jeb.126.1.375

[b47] Kuterbach D. A. & Walcott B. R. Iron-containing cells in the honeybee (*Apis mellifera*). II. Accumulation during development. J. Exp. Biol. 126, 389–401 (1986). 3805999 10.1242/jeb.126.1.389

[b48] Hsu C. Y., Ko F. Y., Li C. W., Fann K. & Lue J. T. Magnetoreception system in honeybees (*Apis mellifera*). PLoS One 2, e395 (2007). 17460762 10.1371/journal.pone.0000395 PMC1851986

[b49] Wu G. R. *et al.* Wing dimorphism and migration in the brown planthopper, *Nilaparvata lugens* Stål. Insect life-cycle polymorphism. Series Entomologica 52, 263–275 (1994)

[b50] Kisimoto R. Synoptic weather conditions inducing long-distance immigration of planthoppers, *Sogatellafurcifera Horvath* and *Nilaparvala lugens* Stål. Ecol. Ent. 1, 95–109 (1976)

[b51] Xu H. J. *et al.* Two insulin receptors determine alternative wing morphs in planthoppers. Nature 519, 464–467 (2015). 25799997 10.1038/nature14286

[b52] Abraçado L. G., Esquivel D. M. S. & Wajnberg E. Solenopsis and magnetic material: Statistical and seasonal studies. Physi. Biol. 6, 10.1088/1478-3975/6/4/046012 (2009) 19887705

[b53] Loper G. M. Influence of age on the fluctuation of iron in the oenocytes of the honey bee (*Apis mellifera*) drones. Apodologie 16, 181–184 (1985)

[b54] Bao Y. X., Zhai B. P. & Cheng X. N. Numerical simulation of the migration parameters of the brown planthopper, *Nilaparvata lugens*(Stål). Sheng Tai Xue Bao 25, 1107–1114 (2005).

[b55] Cheng J. Y. Study on radar observations and trajectory of the autumn migration of brown planthopper. Remote Sens. Environ. 9, 51–56 (1994).

[b56] Kirschvink J. L., Walker M. M. & Diebel C. E. Magnetite-based magnetoreception. Current opinion in neurobiology 11, 462–467 (2001) 11502393 10.1016/s0959-4388(00)00235-x

[b57] Gould J. L. In Magnetite Biomineralization and Magnetoreception in Organism, Vol. 5 *Topics in Geobiology* (eds Kirschvink J. L., Jones D. S. & MacFadden B. J. ) Ch. 12, 257–268 (Springer US, 1985).

[b58] Xue J. *et al.* Genomes of the rice pest brown planthopper and its endosymbionts reveal complex complementary contributions for host adaptation. Genome Biol. 15, 521, 10.1186/s13059-014-0521-0 (2014). 25609551 PMC4269174

[b59] Faivre D. *et al.* Development of cellular magnetic dipoles in magnetotactic bacteria. Biophysical journal 99, 1268–1273, 10.1016/j.bpj.2010.05.034 (2010). 20713012 PMC2920646

[b60] Romeis B. Miikroskopische Technik. (Munich: Oldenbourg-Verlag, 1968).

